# The presence of the gadolinium-based contrast agent depositions in the brain and symptoms of gadolinium neurotoxicity - A systematic review

**DOI:** 10.1371/journal.pone.0171704

**Published:** 2017-02-10

**Authors:** Cyprian Olchowy, Kamil Cebulski, Mateusz Łasecki, Radosław Chaber, Anna Olchowy, Krzysztof Kałwak, Urszula Zaleska-Dorobisz

**Affiliations:** 1 Department of General and Pediatric Radiology, Wroclaw Medical University, Wrocław, Poland; 2 Radiology Students’ Scientific Club, Wrocław Medical University, Wroclaw, Poland Department of General and Pediatric Radiology, Wroclaw Medical University, Wroclaw, Poland; 3 Division of Hematology, Cancer Prevention and Propaedeutics of Rare Diseases, Faculty of Medicine, University of Rzeszow, Rzeszów, Poland; 4 Department of Pediatric Hematology, Oncology and BMT, Wroclaw Medical University, Wrocław, Poland; University of South Florida, UNITED STATES

## Abstract

**Background and purpose:**

Gadolinium based contrast agents (GBCAs) are widely used in magnetic resonance imaging, but recently, high signal intensity in the cerebellum structures was reported after repeated administrations of contrast- enhanced magnetic resonance images. The aim of this systematic review was to investigate the association between increased signal intensity in the dentate nucleus and globus pallidus in the brain and repeated administrations of GBCAs. Additionally, we focused on possible short- and long-term consequences of gadolinium use in those patients.

**Methods:**

Systematic review of retrospective investigations in PubMed and Medline was performed in July 2016. Primary outcomes included the presence of increased signal intensity within the dentate nucleus and globus pallidus on unenhanced T1-weighted MR images in patients following administrations of GBCAs. Two independent reviewers were responsible for search and data extraction.

**Results:**

25 publications satisfied inclusion criteria (19 magnetic resonance images analyses, 3 case reports; 3 autopsy studies). Magnetic resonance images of 1247 patients with increased signal intensity on unenhanced T1-weighted MR images were analyzed as well as tissue specimens from 27 patients. Signal intensity correlated positively with the exposure to GBCAs and was greater after serial administrations of linear nonionic than cyclic contrast agents. Gadolinium was detected in all tissue examinations.

**Conclusions:**

High signal intensity in the dentate nucleus and globus pallidus on unenhanced T1-weighted magnetic resonance images were associated with previous administration of GBCAs. Signal intensity correlated negatively with stability of contrast agents. Clinical significance of gadolinium deposition in the brain remains unclear. There is a strong need for further research to identify type of gadolinium deposited in the brain as well as to gather knowledge about long-term consequences.

## Introduction

Magnetic resonance imaging (MRI) is one of the fastest developing noninvasive diagnostic modalities in medicine. It employs innovative techniques and the newest discoveries, which are safe and effective, but even so it is difficult to avoid pitfalls especially regarding long-term consequences. The safety of gadolinium is now the most frequently discussed topic. This rare paramagnetic metal is widely used in diagnostic imaging due to its high magnetic moment and relatively long magnetic relaxation time. High toxicity of free gadolinium was eliminated by closing free Gd3+ions in organic chelates. [1] Gadolinium based contrast agents (GBCAs) are widely used in medicine since 1988, after Magnevist (Bayer Healthcare Pharmaceuticals) gained the U.S. Food and Drug Administration approval. In 2014 unfortunately, Kanda et al. observed connection between previous gadolinium administrations and high signal intensity in the dentate nucleus and globus pallidus in the human brain independent of renal function. [2] This discovery has drawn attention to the safety of contrast enhanced MRI. The impact of gadolinium on human health remains unknown, although efforts are made to understand the mechanisms of gadolinium toxicity which can help to find therapeutic and preventive solutions for patients presenting signs and symptoms of gadolinium accumulation.[3]

### Aim of the review

To shed more light on recent controversies regarding the use of GBCAs for MRI, we performed a systematic review of the current literature with the aim to investigate the association between increased signal intensity in the dentate nucleus and globus pallidus in the brain of patients with a history of GBCA administrations. Additionally, we focused on possible short- and long-term consequences of gadolinium use.

## Search strategy

This study was done according to the guidelines of systematic reviews PRISMA. [4] We performed a systematic search of PubMed and Medline databases for studies on MRI of the brain, which assessed signal intensity of the dentate nucleus and globus pallidus performed in patients following MRI examinations with GBCAs. The search was carried out in July 2016. The search terms were gadolinium AND (dentate nucleus OR globus pallidus) and their combinations. Additionally, we supplemented the electronic searches by hand searching the bibliographies of relevant articles. Titles and abstracts were screened by two authors in order to exclude duplicates and articles that did not meet the inclusion criteria. All other studies were retrieved for full-text assessment performed by two researchers. Any discrepancies between the two reviewers were resolved by consensus, with a third reviewer consulted when necessary.

### Inclusion and exclusion criteria

Original studies, which investigated signal intensity in dentate nucleus and globus pallidus examined in patients who had undergone repeated GBCA-enhanced magnetic resonance (MR) were included in the analysis. We excluded studies with patients, in whom increased signal intensity in those areas of the brain may have appeared due to other reason than gadolinium deposition such as underlying Wilson disease, Rendu-Osler-Weber disease, manganese toxicity, calcification, neurofibromatosis, or a history of total parenteral nutrition. Studies including patients having hepatic or renal dysfunction as well as cases reports were analyzed for information about side effects and toxicity related to gadolinium depositions in the brain. Letters, replies, editorials, review articles, conference abstracts and animal studies were excluded.

## Results

**Fig 1:**
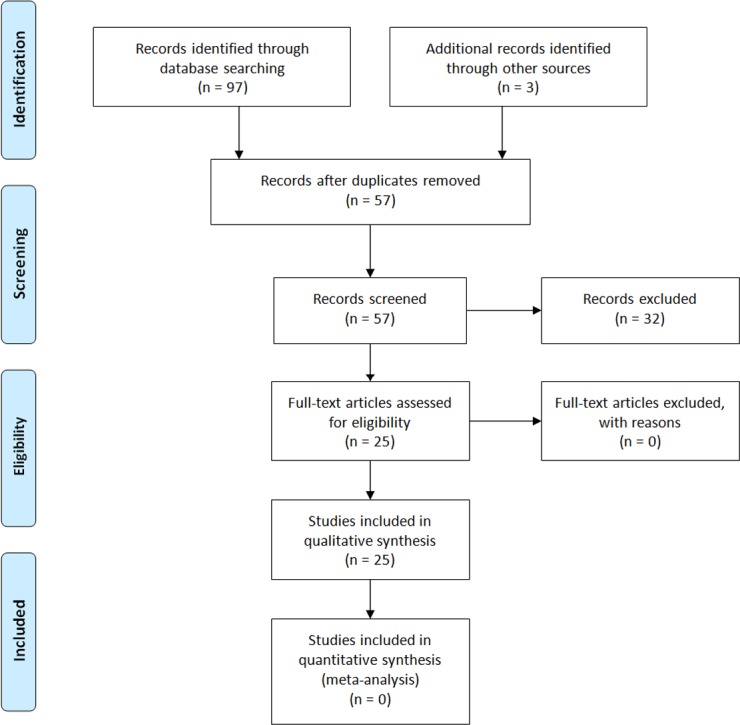
Flow Diagram based on PRISMA Statement [4].

The electronic search strategy identified 54 publications; three additional papers were retrieved by hand-search. Of this number, 25 studies satisfied all inclusion and exclusion criteria for this review. Among included studies, there were 19 original studies of magnetic resonance images, 3 case reports, and 3 autopsy studies. In total, 1247 patients with increased signal intensity on unenhanced T1-weighted MR images as well as tissue specimens from 27 patients were analyzed. All the studies included into analysis were available and written in English. Process of study inclusion and exclusion is presented in Fig 1. Table 1 includes the list of analyzed publications with a summary of main findings.

**Table 1:** Publications included in the systematic review.

	Study	Study groups	Administrations of GBCA	Contrast agent	Remarks
	**Original studies**				
1	Adin et al. 2015 [42]	• Control = 54 • Increased SI = 103	• 0 • 2–60; median 18	Predominance of gadopentetate dimeglumine (Magnevist)	No significant difference in SI between Magnevist and non-Magnevist group
2	Cao et al I 2016 [37]	• On hemodialysis = 25 • Control on hemodialysis = 13 • Near-normal renal function = 25 • Control near normal renal function = 13	• 1.8 ± 1 • 0 • 1.8 ± 1 • 0	linear GBCA	The percentage of patients with increased SI in DN post-GBCA was significantly greater in relation to pre-GBCA only in the group on hemodialysis.
3	Cao et al II 2016 [10]	• Gadobutrol group = 25 • Gadopentetate dimeglumine = 25	• 7.8 ± 2.4 (6–16) • 12.1 ± 5.2 (6–23)	gadobutrol and gadopentetate dimeglumine	Significant increase in SI in gadopentetate dimeglumine group and insignificant in gadobutrol group.
4	Errante 2014 [30]	• MS group = 38 • Brain metastases group = 37	• 2–14 • 2–21	gadodiamide (Omniscan)	DNP were significantly higher between the first and the last scan in both study groups in patient with 6 or more examinations.
5	Flood 2016 [25]	• GBCA-naive control subjects = 57 • GBCA-exposed patients = 30 • Pre- and post-GBCA comparison = 16	• 0 • 5.9 ± 2.7 • 5.8 ± 2.1	gadopentetate dimeglumine (Magnevist)	SI in DN was significantly higher in the GBCA-exposed group than the GBCA-naive group.
6	Hu 2016 [19]	• Control group = 21 • GBCA group = 21	• 0 • 5–37	gadopentetate dimeglumine (Magnevist)	Significant increase in the ND-to- corpus callosum genu ratio and the GP-to- corpus callosum genu ratio between the first and most recent GBCA exam.
7	Kanda 2014 [2]	• contrast-enhanced MR subgroup = 19 • unenhanced MR subgroup = 16	• 7.1 (6–12) • 0	gadopentetate dimeglumine (Magnevist) gadodiamide (Omniscan)	DNP and GPT ratio was correlated with the number of previous contrast-enhanced MR scan.
8	Kanda 2015 [9]	• Magnevist group = 23 • ProHance group = 36 • Both agenst = 14 • Control = 54	• median 2; max 11 • median 2; max 15 • median 2; max 5 • 0	gadopentetate dimeglumine (Magnevist) gadoteridol (ProHance)	Only the DN-to-cerebellum ratio was significantly associated with linear GBCA.
9	Kromrey 2016 [18]	• Gadobutrol group = 271 • Control group = 116	• 1 • 0	gadobutrol	After 5-year follow-up, no significant differences in SI were observed between gadobutrol group and controls as well as between baseline and follow-up measurement.
10	Quattrocchi 2015 [14]	• group A = 10 • group B = 28 • group C = 8	• 1 • 1–5 • >6	gadodiamide	A significant increase of DNP was between the first and the last MRI in group C.
11	Radbruch I 2015 [12]	study group = 30	7.3 ± 3.1	gadobutrol	No SI increase in the DN and GP was found after serial applications gadobutrol.
12	Radbruch II 2015 [3]	• Magnevist group = 50 • Dotarem group = 50	• 7.32 ± 1.83 • 7.06 ± 1.20	gadopentetate dimeglumine (Magnevist), gadoterate meglumine (Dotarem)	In the linear GBCA group, the mean difference in SI of DNP between the last and first examinations was significantly larger than 0, while in the macrocyclic GBCA group was not significant.
13	Ramalho I 2015 [17]	• Omniscan group = 23 • MultiHance group = 46	• 5.0 ± 2.4 (3–11) • 4.6 ± 2.1 (3–11)	gadodiamide (Omniscan) gadobenate dimeglumine (MultiHance)	GPT and DN-to- middle cerebellar peduncle ratio increased significantly over time with multiple administrations of a linear nonionic GBCA (gadodiamide) but did not increase with serial applications of a linear ionic GBCA (gadobenate dimeglumine).
14	Ramalho II 2016 [21]	• gadobenate dimeglumine = 44 • previous gadodiamide and current gadobenate dimeglumine = 18	• 4.5 ± 2.0 (3–11) • 5.9 ± 2.7 (3–11) • 5.5 ± 1.9 (3–10)	gadodiamide (Omniscan) gadobenate dimeglumine (MultiHance)	Significant increase in DN-to- middle cerebellar peduncle ratio appeared after gadodiamide injections. It was insignificantly higher in the group receiving both GBCAs.
15	Ramalho II 2016 [44]	T1-weighted spin-echo and 3D MPRAGE = 18	4.78 ± 2.51 (2–10)	gadodiamide (Omniscan)	The differences in DN-to- middle cerebellar peduncle ratio between the 2 sequences for both baseline and last examination were statistically significant, but the change in ratio with time was not.
16	Stojanov 2015 [31]	relapsing-remitting MS group = 58	4.74±0.72 (4–6)	gadobutrol (Gadovist)	DNP, but not GPT, showed a significant correlation with the number of administered doses of GBCA.
17	Tanaka 2016 [45]	• MS group = 21 • neuromyelitis optica spectrum disorder group = 6	at least 10	linear	Number of GBCA doses correlated positively with signal intensity increase in 81% of MS patients and in 33.3% of NMOsd patients.
18	Tedeschi 2016 [15]	relapsing-remitting MS group = 74	6.0 ± 3.8 (1–15)	gadopentetate dimeglumine,gadobutrol	Previous gadolinium administrations correlate with DN T1 relaxometry and are linked to linear gadolinium chelates.
19	Weberling 2015 [11]	study group = 50	7.7 ± 3.2 (5/15)	gadobenate dimeglumine	The DNP and DN-to-cerebrospinal fluid ratio difference between the first and the last MRI was significantly larger than 0.
	**Case reports**				
20	Barbieri 2016 [38]	3 cases patients with impaired renal function and vascular calcification; two of them had NSF.		Gadoteridol, Gadodiamide	Increased SI within DN and GP after exposure to relatively low doses of linear GBCAs
21	Roberts 2016 [24]	A patient with a clival chordoma.		gadopentetate dimeglumine (Magnevist)	Subtle hyperintensity within DN appeared after 4 doses of MRI contrast and after sixth dose hyperintensity was noted within both DN and GP bilaterally.
22	Miller 2015 [46]	A pediatric patient.	35	Linear GBCA	Progressive increases were the most evident in DN, DP, and the thalamus.
	**Autopsy examinations**				
23	McDonald 2015 [20]	• GBCA group = 13 • Control group = 10	At least 4	gadodiamide	DN was associated with the greatest dose-dependent change in T1-weighted signal intensity, whereas the pons demonstrated the lowest overall absolute change in T1-weighted signal intensity with gadolinium. All patients exposed to multiple doses of a GBCA had elevated levels of elemental gadolinium in the four prescribed neuroanatomic regions.
24	Kanda 2015 [27]	• GBCA group = 5 • Non-GBCA group = 5	At least 2	• gadopentetate dimeglumine (Magnevist) • gadodiamide (Omniscan) • gadoteridol (ProHance)	Gadolinium was detected in all specimens in the GBCA group and in some specimens in the non-GBCA group; however, the gadolinium concentration was significantly higher in the GBCA group in each region.
25	Murata 2016 [28]	• gadoteridol group = 5 • gadobutrol group = 2 • gadobenate group = 1 • gadoxetate group = 1 • control group = 9		• gadoteridol (ProHance) • gadobutrol (Gadovist) • gadobenate (MultiHance) • gadoxetate (Eovist)	Gadolinium deposition in normal brain and bone tissue occurred with macrocyclic and linear GBCAs in patients with normal renal function.

Legend: SI, signal intensity; GBCA, gadolinium based contrast agent; DN, dentate nucleus; GB, globus pallidus; DNP, dentate nucleus-to-pons ration; GPT, globus pallidus-to-thalamus ratio; MS, multiple sclerosis; NMOsd, neuromyelitis optica spectrum disorder group

### Association between increased signal intensity in the deep cerebellar nuclei and previous administrations of GBCAs

Gadolinium is a heavy metal with unique paramagnetic properties. Due to seven unpaired electrons, it has the best relativity of the other lanthanide ions. Gadolinium(III) ions (GD3+) are toxic, but bound to a ligand, they can remain chelated in the body and are excreted intact. [1] The list of available GBCAs is presented in Table 2.

**Table 2:** GBCAs approved for clinical use.

**Nonionic Linear)**	**Ionic Linear**
• Omniscan (gadodiamide) • OptiMARK (gadoversetamide	• Ablavar (gadofosveset trisodium) • Eovist (gadoxetate disodium) • Magnevist (gadopentetate dimeglumine) • MultiHance (gadobenate dimeglumine)
**Nonionic macrocyclic**	**Ionic macrocyclic**
• ProHance (gadoteridol) • Gadavist (gadobutrol)	• Dotarem (gadoterate meglumine)

Increased signal intensity within the dentate nucleus and globus pallidus on unenhanced T1-weighted MR images was observed by many radiologists, but its association with gadolinium was not obvious [5–7]. Only in 2013, Kanda et al. noticed the important connection between changes visible on MRI scans and a history of multiple administrations of gadolinium-based contrast material in patients with normal renal function. This research team selected 19 patients who had undergone at least 6 contrast- enhanced MR examinations with gadopentetate dimeglutamine (Magnevist) or gadodiamide (Omniscan) and 16 patients who had at least 6 MR scans without contrast administration. Analysis of the data revealed a positive correlation between the number of previous gadolinium-based contrast material administrations and both the dentate nucleus-to-pons ratio and globus pallidus-to-thalamus ratio. [2] Since this discovery, scientists from around the world began the race to provide more information about association between gadolinium and signal intensity changes in the brain.

Different molecule structure due to bonding gadolinium to various ligands results in differences in physicochemical properties. In linear agents, Gd3+ is combined with open-chain ligands, while in macrocyclic agents, the gadolinium ion is caged inside the cavity of organic structure, which resembles a ring. [8] The structural difference between macrocyclic and linear open-chain chelates mainly has an impact on the stability of the gadolinium chelate. Kanda’s team aimed to compare the effect of administration of linear and macrocyclic gadolinium chelates. In the study group, hyperintensity in the dentate nucleus was found in 9 patients, out of which 7 were given only linear chelate GBCA. The dentate nucleus-to-cerebellum ratio was significantly associated with linear GBCAs. [9] Similar approach was used by Cao et al. who compared two groups of patients: those who received gadobutrol (macrocyclic GBCA) with those who received gadopentetate dimeglumine (linear GBCA). In gadopentetate dimeglumine group, signal intensity measured in dentate nucleus increased significantly after six administrations, while in gadobutrol group signal increase was not significant. [10] Other researchers also reported no signal intensity increase in the dentate nucleus and globus pallidus after administration of macrocyclic GBCAs and significant signal increase after repeated administration of linear GBCAs in cancer patients. [11–14] Tedeschi linked changes in T1 relaxometry of dentate nuclei observed in patients with relapsing-remitting multiple sclerosis to the number of previous GBCA administrations. T1-hyperintensity was highly significant for linear GBCA. [15]

Transmetalation of gadolinium chelates and the rate of the gadolinium ions release from chelates in the presence of Zn2+ at pH 7.4 decreases relaxation rates. On this basis, 3 classes of gadolinium contrast agents were determined using a long-term index which is equal to the ratio of the paramagnetic relaxation rates after 50 h. [8,16] Macrocyclic chelates with very high kinetic inertia stability have a long-term index exceeding 0.95, ionic linear open-chain chelates, which have moderate kinetic inertia, have long-term index ranging from 0.49 to 0.85 and nonionic linear open-chain chelates characterized by poor kinetic stability and the highest degree of dechelation have long-term index below 0.3. [8] Clinical comparison between two types of linear GBCAs (ionic and non-ionic) was conducted by Ramalho et al. The study protocol was based on comparison of signal intensity in globus pallidus and dentate nucleus between linear nonionic gadodiamide (Omniscan) and linear ionic gadobenate dimeglumine (MultiHance). After average of 5 injections of gadolinium based contrast, signal intensity ratios based on measurement within dentate nucleus and middle cerebellar peduncle as well as globus pallidus and thalamus increased significantly only in the group which received gadodiamide having the worst long-term index. [17] Radbruch et al. reported that in case of macrocyclic GBCAs, the difference in signal intensity in similar area was not significant even after average of 7 examinations with gadoterate meglumine and gadobutrol. [12,13]

The long-term effect of a single injection of gadobutrol was examined based on SHIP database conducted as a prospective population-based cohort study. After 5-year follow-up, no significant difference in signal intensity was observed between gadobutrol group and controls as well as between baseline and follow-up measurement. Signal intensity was measured within thalamus, pallidum, pons, dentate nucleus as well as white matter and gray matter. The study shows that gadobutrol does not lead to a measurable increase in signal intensity in neuronal structures when applied even at a 1.5 times higher dose than generally required. [18] On the contrary, Hu et al. reported visible changes as early as on the second examination carried out with gadopentetate dimeglumine (ionic linear GBCA) in two pediatric patients. [19]

Investigators are interested in not only when the changes appear, but also whether they can resolve. Usually, increased signal intensity within brain structures were reported after repeated administrations of GBCAs of at least four times in case of linear contrast agents [12,20], but in case of macrocyclic ones, exposure to six applications resulted in insignificant increase in signal intensity [10] Ramalho et al. showed that injections of a contrast agent characterized by poor stability in the past results in signal increase even after using more stable contrasts in subsequent examinations, which draws attention to potential potentiating effect of unstable agents. [21] Birka et al. described a case of patient with nephrogenic systemic fibrosis; detectable amounts of gadolinium-phosphate deposits were found after 11 years post administration of GBCA. [22] None of the authors reported decrease in signal intensity over time at the time of writing our paper (by July 2016); however, during a revision of the manuscript, Radbruch et al. reported a decrease in preexisting signal hyperintensity after changing from linear to macrocyclic GBCA. It was suggested that some washout may exist. [23]

Many researchers reported similar changes within brain structures after gadolinium enhanced MRI scans in pediatric patients. In the Hu’s et al. study, the changes, both in the dentate nucleus ratio and the globus pallidus ratio, were significant when compared between the first and most recent GBCA examination. Investigators draw the attention to the fact that hyperintensity either in the dentate nucleus or the globus pallidus was visually noticed within the first 10 GBCA exams in all patients and in some patient even after 2 administrations of contrast, which may indicate greater susceptibility in children. The correlation analysis revealed no significant associations between signal intensity ratios and both the number of examinations or the total volume of GBCA administered. [19] Roberts et al. described the case of a girl with a clival chordoma in whom subtle hyperintensity appeared after fourth administration of linear GBCA. [24] Flood et al. showed that signal intensity within the dentate nucleus was significantly higher in the GBCA-exposed group than the GBCA-naive group. Additionally, signal intensity comparison before and after repeated GBCA exposures showed significant increase within dentate nucleus, while within the globus pallidus signal intensity change remained insignificant. Additionally, the dentate nucleus-to-pons ratio was significantly correlated with the total cumulative gadolinium dose, but insignificantly with the total number of contrast-enhanced examinations. [25]

### Evidence of gadolinium deposition in the brain

An increase of relative signal intensity in the dentate nucleus and the globus pallidus on unenhanced T1-weighted MR images following administration of GBCAs was linked to gadolinium administration, but it was generally believed that the gadolinium chelates do not diffuse through plasma membranes and do not cross the intact blood-brain barrier. [26]

The first report about gadolinium deposition in the brain was published in 2011. Sanyal et al. analyzed tissues from an autopsy case with verified advanced nephrogenic systemic fibrosis and detected insoluble Gd-phosphate deposits in many organs including cerebellum; however, gadolinium depositions were found only in the perivascular glial cells. [26] McDolnald et al. aimed to examine if signal hyperintensity within dentate nucleus and globus pallidus visible on T1-weighted MR images after repeated administrations of GBCAs is associated with gadolinium deposition in neuronal tissues. They found elevated levels of elemental gadolinium in the dentate nucleus, pons, globus pallidus, and thalamus in all patients exposed to multiple doses of a linear GBCA, whereas control patients demonstrated undetectable levels of elemental gadolinium. Gadolinium concentration was strongly correlated with a cumulative gadolinium dose. Examinations showed major gadolinium deposits in the endothelial walls and small fraction of gadolinium deposited in the neural tissue interstitium, which suggests crossing intact blood-brain barrier. [20] Due to technical difficulties, Kanda et al. analyzed smaller than in McDonald’s study groups of deceased patients who had been given different GBCAs. The gadolinium concentration in dentate nucleus, globus pallidus, cerebellar white matter, frontal lobe cortex, and frontal lobe white matter was significantly higher in the GBCA group than in non-GBCA group. Detection of gadolinium in the samples from non-GBCA group was explained by contamination. [27] Murata et al. examined brain specimens in patients who had received either linear of macrocyclic GBCAs. Among the tested agents, they found the lowest gadolinium deposition ratio in brain tissue for the macrocyclic agent gadoteridol. [28] The greatest limitation of brain autopsy studies is that they were not able to detect a specific form of gadolinium.

In the view of current discussion about form of gadolinium deposition, the case reported by Birka et al. brings additional information; though, it concerns a patient with nephrogenic systemic fibrosis. The investigators were able to determine GdPO4 and gadoteridol from skin specimens from a patient who received one administration of a linear GBCA (gadopentetate) and one of a macrocyclic GBCA (gadoteridol) given 11 and 8 years before tissue examination. Chelated forms of gadolinium were not found. [22]

### Multiple sclerosis

The first observations of increased signal intensity in dentate nuclei were linked to the secondary progressive disease subtype of multiple sclerosis and to increased clinical disability, lesion load, and brain atrophy. [5] Post-inflammatory changes are common in the brain of patients suffering from multiple sclerosis and may resemble post-GBCA administration hyperintensity of the dentate nucleus visible on unenhanced T1-weighted images. Diagnosis of multiple sclerosis as well as assessment of new lesions in the brain requires repeated contrast enhanced MR examinations. Contrast enhancement on post-gadolinium T1-weighted imaging help to differentiate acute and chronic lesions. [29] Errante et al. proved that signal increase in patients with multiple sclerosis depends not only on underlying primary disease but also on the number of previously performed gadolinium- enhanced MR examinations. According to their study, T1 signal intensity of the dentate nucleus in patients with at least 6 enhanced MRI scans was significantly higher than those with less than 6 scans. [30] Stojanov et al. focus on examinations performed in patients with relapsing-remitting multiple sclerosis who had undergo several MR scans with gadobutrol, which is macrocyclic non-ionic GBCA, characterized by better stability than used by Errante et al. open chain gadodiamide. [31] Dentate nucleus-to-pons ratio increased significantly after the last examination but the change of globus pallidus-to-thalamus ratio was insignificant. Those results were obtained after average of 4.74 gadolinium-based contrast administrations, which is quite early comparing with study groups excluding patients with multiple sclerosis. Later, other researchers questioned Stojanov’s team outcomes because of methodological limitations of the study. [11,32]

### Renal insufficiency

As early as in 2006, Grobner informed about an association between magnetic resonance angiography with gadopentetate dimeglumine as a contrast agent performed in patients with end-stage renal disease and the development of nephrogenic systemic fibrosis. [33] Cabot et al. reported a patient who died of nephrogenic systemic fibrosis. High levels of gadolinium were found in all biopsied tissues; unfortunately, the brain tissue samples were then not collected for autopsy. [34] A year later Sanyal et al., reported the presence of insoluble gadolinium-phosphate deposits also in the cerebellum; however, they were detected only in the perivascular glial cells. [26]

One of the factors that contribute to the pathogenesis of nephrogenic systemic fibrosis is the slow excretion of GBCAs in those patients allowing the lower stability gadolinium chelates to dissociate and release toxic Gd3+. Additionally, gadodiamide can activate elevated fibroblast growth and elevated levels of hyaluronan synthesis [35], which are responsible for clinical symptoms of nephrogenic systemic fibrosis.

Patients with renal failure are exposed to prolonged effect of injected GBCAs because prompt elimination though renal excretion is impossible in those patients. Impairment of renal function is than a risk factor for deposition of gadolinium in tissues, including the brain. [36] As a result, signal intensity in dentate nucleus on unenhanced T1-weighted images was greater in hemodialysis patients than in patients with normal renal function after repeated administrations of GBCAs. [37] Additionally, Barbieri et al. described three cases with impaired renal function and vascular calcification, which presented with signal hyperintensity within the dentate nucleus and the globus pallidus on unenhanced T1-weighted MR images after exposure to relatively low doses of linear GBCAs. [38] Those reports suggest that impaired renal function accelerates the rate of gadolinium accumulation in the brain.

Patients with a low estimated glomerular filtration rate are at higher risk of complications related to GBCA administration than those with normal renal function. Currently, the Contrast Media Safety Committee of the European Society of Urogenital Radiology recommends administering the smallest required amount of macrocyclic GBCAs, keeping at least weekly break between injections and planning an additional dialysis session in patients on dialysis. [39] Patients with impaired renal function should be carefully monitored, as they are prone to known and unknown adverse reactions of gadolinium.

### Brain irradiation

Radiation-related injury to the brain may result in calcifications, which are visible as an increase of magnetic resonance signal intensity. [40] Post-radiation changes may give non-specific hyperintense signal changes which are similar to many other conditions affecting the dentate nuclei [41]; therefore, patients who had undergone brain irradiation are commonly excluded from the studies examining the presence of hyperintense signal changes in relation to previous gadolinium exposure. In our review, Adin’s at al. study included 129 patients with a history of brain radiotherapy among other. Of that group, specific radiation exposures of the dentate nuclei were confirmed for 108 subjects. Their analysis revealed that in the group with increased signal intensity, the total exposure of the brain was lower than in the group without hyperintensity, although the groups did not differ significantly. [42] Adin’s team report is in line with Kanda et al. findings who reported that brain irradiation had no impact on signal change. [2]

### Neurotoxicity of GBCAs

Gadolinium chelates are considered safe because of their efficient renal excretion from the body in an unchanged form. However, GBCAs have different stability, and therefore some agents are more prone for dechelation than others are. Toxicity of gadolinium is associated with similarity between gadolinium and calcium ions, but refers exceptionally to free ions. Gd3+ can compete with Ca2+ and act as an inorganic blocker of voltage-gated calcium channels with all negative consequences in many sites of the human body. [1] Despite extensive research, little is known about possible consequences of gadolinium brain depositions. Neither mechanism responsible for collection of gadolinium in dentate nucleus and globus pallidus nor the form of deposited gadolinium has been discovered.

The analysis of clinical events in 16 dialysis patients performed by Cao et al. allowed for comparison of their frequency during a period of a month before with a month after each exposure to linear GBCA. All neurological symptoms described during the 30 days after GBCA administration such as migraine headache, loss of consciousness, memory loss, falling, lightheadedness, and ataxia, occurred also before MRI with GBCA; therefore, they cannot be related to gadolinium toxicity. [37] Barbieri et al. described three patients with impaired renal function and vascular calcifications who presented with signal hyperintensity characteristic for gadolinium brain depositions. All of them suffered from transient signs of neurological disorders of undetermined cause, but the association of those symptoms with gadolinium toxicity remains unclear. [38]

There are several patient advocacy groups, who gather people suffering from nephrogenic systemic fibrosis and other symptoms related to GBCAs administration. Recently, a self-report survey among patients, who attributed their symptoms to previous GBCA administrations, was published. Respondents (n = 50) received intravenous gadolinium contrast with an average of 4.2 doses (range 1–23). Two-thirds of them experienced those symptoms immediately following GBCA-enchanced examination. Bone/joint pain as well as head/neck problems such as headache, vision change, and hearing change were mentioned the most frequently; they occurred in more than 75% of questionnaires. About 60% of respondents complained about dermatologic changes. [43] The study has a long list of limitations, but constitutes the first description of symptoms, which may be related to gadolinium toxicity.

### Technical considerations

The number of studies on the effect of using various GBCAs on the brain has been rapidly increasing since the first report about association of previous repeated GBCAs and hyperintensity of dentate nucleus and globus pallidus. [2] The level of knowledge increases, but when analyzing data, it seems that some researchers report incompatible findings affected by unintended limitations. First, qualitative and quantitative signal-intensity measurement methods are used together in many studies. Many MRI protocols employed sequences such as T1-weighted spin-echo [10,17,42], T1 MPRAGE [42], T1 FLAIR [30,42], T1-weighted 3D MPRAGE [18,25] and FLASH [11] alone or in combination. Ramalho et al. concluded that T1-weighted spin-echo and MPRAGE sequences should not be used interchangeably for qualitative or quantitative T1 signal-intensity analysis of the dentate nucleus in patients who undergo several gadolinium-enhanced MR examinations as those methods result in statistically significant differences. [44]

Next, as those studies are retrospective in nature, it is sometimes difficult to retrieve information or to collect proper study population. Studies present outcomes of different number of previous exposure to GBCAs administered at different time intervals; thus, heterogeneity of study samples is a potential source of bias. Many of the reviewed studies included cancer patients who had undergone radiation therapy [9–13], some included patients with impaired renal function (estimated glomerular filtration rate <60 mL/min per 1.73 m2) [12] or increased liver serum parameters [11–13] among others.

### Conclusions

Literature review confirms that increased signal intensity in the dentate nucleus and globus pallidus on unenhanced T1-weighted MR images is associated with previous administrations of GBCAs, predominantly linear, and corresponds with the concentration of gadolinium in the brain tissue.

Despite of rapidly growing number of published papers, the level of knowledge about gadolinium depositions in the brain and their clinical significance remains insufficient; therefore, it seems to be reasonable to choose the most stable types of GBCAs and avoid higher doses especially in children and young patients even with normal renal function.

There is a strong need for further research to shed light on the nature of gadolinium deposition in the brain, their effect on the brain tissue functioning and occurrence of long-lasting adverse reaction. Multidisciplinary approach including participation of radiologists, neurologists, psychiatrists, and biologists who could evaluate the clinical significance of gadolinium depositions in the brain in prospective projects encompassing pediatric patients are required.

## Supporting information

S1 File Prisma Checklist. (DOC)
